# Cerebral air embolism via port catheter and endoscopic retrograde cholangio-pancreatography

**DOI:** 10.1186/2193-1801-2-477

**Published:** 2013-09-22

**Authors:** Ivana Vachalová, Stefan Ernst, Irina Vynogradova, Silke Wöhrmann, Josef G Heckmann

**Affiliations:** Department of Neurology, Municipal Hospital Landshut, Robert-Koch Str. 1, 84034 Landshut, Germany; Department of Radiology, Municipal Hospital Landshut, Landshut, Germany; Department of Internal Medicine II, Municipal Hospital Landshut, Landshut, Germany

**Keywords:** Cerebral air embolism, Cranial computed tomography, Port catheter, Endoscopic retrograde cholangio-pancreatography, Esophagogastroduodenoscopy

## Abstract

**Background:**

Cerebral air embolism (CAE) is a critical clinical condition necessitating rapid diagnosis and therapeutic measures.

**Methods:**

The authors describe two patients with lethal CAE.

**Results:**

An 81-year-old man rapidly developed coma with tetraplegia. CT-scan revealed prominent CAE whereby the entry of the air was via a port catheter for parenteral nutrition. A 45-year-old man with severe alcohol-toxic multiple organ damage needed endoscopic retrograde cholangio-pancreatography (ERCP) and a second esophagogastroscopy. After an epileptic seizure, the CT-scan of the brain showed small amounts of cerebral air in the posterior right hemisphere and in the sagittal superior sinus. Despite critical care the patient died.

**Conclusion:**

CAE is a neurocritical emergency case. Early CT-scan of the brain can detect air, guide further therapy, and contribute to the assessment of the prognosis.

## Introduction

Cerebral air embolism (CAE) is a critical clinical condition necessitating a rapid diagnosis and therapeutic measures. Often, CAE is associated with insertion, dysfunction, or disconnection of the central venous line (Heckmann et al. 2000; Han et al. 2010). Further causes are spontaneous or traumatic fistulas between air containing organs and the vascular system, surgery, and barotrauma (O’Dowd 2013). In an emergency situation, imaging procedures may give crucial clues to the diagnosis of an air associated disorder such as CAE or others such as pneumatocephalus (Heckmann and Ganslandt 2004; Nern et al. 2012). Herein we report two patients with fatal cerebral air embolism, associated with an embolism via port catheter and endoscopic retrograde cholangio-pancreatography (ERCP).

## Case reports

### Case 1

**Figure 1:**
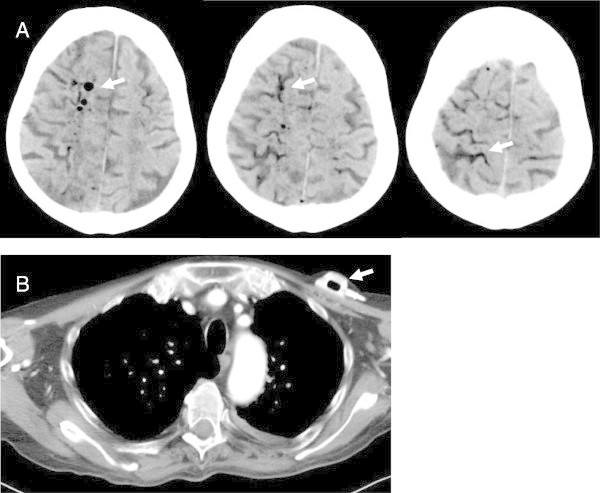
**CT in cerebral air embolism via port catheter.** Panel **A**, native axial CT-scan of the brain shows inclusion of air (arrow) within the subarachnoidal spaces more prominent in the right hemisphere. Panel **B**, CT-thorax shows air in the port catheter (arrow) which is used for parenteral nutrition under home care conditions.

An 81-year old man needed parenteral nutrition via a port catheter after numerous complex bowel surgeries including hemicolectomy and resection of sigma and parts of small bowel due to diverticulitis. Due to comorbidity and later developing dementia he received comprehensive home care. Application of his nutrition was performed by lay personnel. The patient was admitted to the emergency department after the rapid onset of coma with tetraplegia. The initial CT-scan of the brain revealed a cerebral air embolism, more visible in the right hemisphere (Figure 1A). In a CT-scan of the thorax which was performed due to depression of the circulation, there were no signs of a pulmonary embolism or aortic dissection. The port catheter was filled prominently with air (Figure 1B). The clinical condition worsened. According to the patient’s and his relatives’ will, treatment on the intensive care unit including mechanical ventilation was omitted and the patient died two days later. An autopsy was not performed.

### Case 2

**Figure 2:**
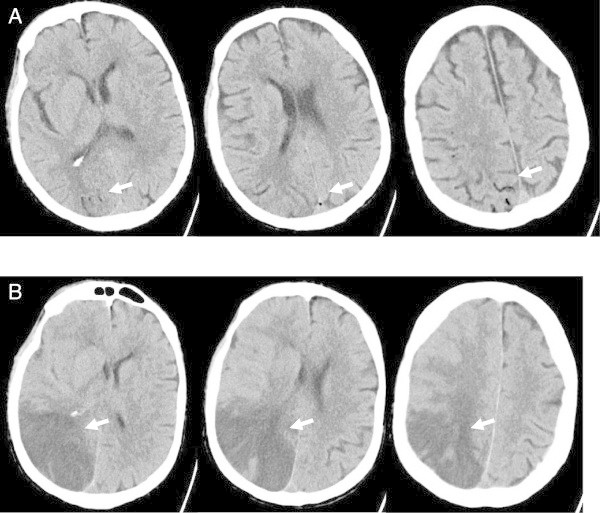
**CT in cerebral air embolism via ERCP.** Panel **A**, native axial CT-scan of the brain shows a small amount of air accumulation in the right posterior subarachnoidal space (arrow) and in the superior sagittal venous sinus (arrow). Panel **B**, native axial CT-scan of the brain two days later shows severe infarction (arrow) predominantly in the right posterior hemisphere with malignant brain swelling.

A 45-year old man with long standing severe alcohol-toxic multiple organ damage suffered from acute bacterial cholangitis due to stenosis of the common bile duct. In addition a space occupying lesion of the pancreas was suspected to be malignant. Therefore an endoscopic retrograde cholangio-pancreatography (ERCP) was performed which revealed a stenosis of the common bile duct and obliteration by additional numerous concrements. Via papillotomy the concrements were removed and a double pigtail catheter was inserted into the common bile duct. A day later, a second esophagogastroscopy was performed due to stenosis of the duodenal bulb. In the esophagus signs of reflux associated esophagitis were found. The etiology of the duodenal stenosis, however, could not be clarified, in the stomach there were no erosions. Shortly after the procedure, the patient had an epileptic seizure. The CT-scan of the brain at this time showed cerebral air in the posterior right hemisphere and isolated air bubbles in the posterior sinus sagittalis superior (Figure 2A). The clinical condition deteriorated rapidly to coma. Despite treatment on the intensive care unit, a malignant space occupying cerebral ischemia developed (Figure 2B). Due to a disastrous coagulation disorder and septic conditions, hemicraniectomy and hypothermia could not be performed and the patient died two days later. An autopsy showed a malignant predominately right-sided brain infarction, acute and chronic pancreatitis, acute erosive cholangitis, toxic liver cirrhosis, and signs of severe sepsis. In addition portal vein thrombosis and inflammatory changes of the surrounding vessels were detected.

## Discussion

In CAE, diagnosis is known to be difficult and to depend on clinical experience (Bäuerle et al. 2013). The patient’s history combined with the clinical suspicion of the embolism due to the initial neurological findings and the direct temporal relation of potentially emboligenic procedures are the most important diagnostic clues (Muth and Shank 2000). Early in the course of the disease, air emboli can be detected by computed tomography (CT) (Valentino et al. 2003). In both reported patients, air could be verified without doubt, as imaging by CT was performed early after the accident. Despite immediate critical care support the course of the disease was lethal in both cases. Because a do-not-resuscitate order existed for patient 1, mechanical ventilation and resuscitation were omitted. In patient 2 intensive care unit therapy was performed. Hyperbaric oxygen therapy (HBO) was desired as it is recommended, in particular within the first 6 h after the accident (Muth and Shank 2000; van Hulst et al. 2003; O’Dowd 2013). However HBO was not available in our hospital at this time and transport to the next HBO facility was considered to be high risk and of questionable benefit due to the patient’s global clinical condition. Recently therapeutic hypothermia was successfully reported in CAE (Bäuerle et al. 2013). However, due to septic conditions and a severe coagulation disorder in patient 2, hypothermia and even hemicraniectomy for decompression were judged to be unreasonable.

As reported in the literature, in CAE the right hemisphere seems to be more affected due to the course of the right brachiocephalic trunc which is the first main artery rising from the aortic trunc (Heckmann et al. 2000). In both reported cases, the right hemisphere was more severely damaged supporting the hypothesis that venous embolism transits into the arterial system and primarily affects the right-sided great vessels from the aortic branch.

Pathophysiologically in patient 1, a mechanism comparable to CAE associated with central venous lines was assumed (Heckmann et al. 2000; Han et al. 2010)). Air entered the venous system in this case by the venous port which had been inserted a year earlier with access to the left cephalic vein. Despite no documentation of a right-left shunt via a persistent foramen ovale, shunts from the venous system to the arterial system are quite frequent occurring via the lung (Han et al. 2010).

In patient 2 the pathophysiology is not as clear. As in earlier reports on ERCP or esophagogastroduodenoscopy-associated CAE, it is speculated that inflammation of the bile duct and its surrounding veins, previous intervention of the bile duct system, and insufflation of air during endoscopy may have contributed to air entering the vascular system either via portal and liver veins or via mucosal disruptions (Pandurangadu et al. 2012; Nern et al. 2012; Koster et al. 2012). If air entered the arterial cerebral circulation, gas bubbles obstruct the cerebral vessels and cause distal ischemia (Muth and Shank 2000) via a complex cascade of damage (van Hulst et al. 2003) which resembles damage resulting from hypoxic brain injury (Bäuerle et al. 2013).

The reported case vignettes are remarkable for two reasons. First, a lethal cerebral air embolism associated with the use of a port catheter under home care conditions has hitherto not been reported according to a literature search. Unfortunately, the exact circumstances which led to the air entry into the port catheter could not be resolved. As a rule, however, a port catheter should be handled with the same caution as a central venous line. Second, a lethal cerebral air embolism in association with ERCP or even esophagogastroscopy is sparsely reported. Including our case and reviews from 2010 and 2012, 11 cases are documented in the literature (Finsterer et al. 2010; Nern et al. 2012). Even the finding of air inclusions within the superficial cortex and the superior sagittal sinus in our second patient is quite unique. Such a finding has been reported earlier in only two patients with ERCP associated CAE (Nern et al. 2012).

## Conclusion

CAE is a neurocritical emergency case which can occur as a complication of numerous invasive medical procedures including care of a port catheter or during ERCP or esophagogastroduodenoscopy as observed in our patients (van Hulst et al. 2003; Muth and Shank 2000; Nern et al. 2012). CT-scan is the primary imaging procedure enabling detection of air particularly in the early course of disease. In addition, CT findings will guide further therapy and contribute to the assessment of the prognosis. These two unusual cases are reported to inspire and excite our colleagues further regarding this topic of neurocritical care.

## Consent

The relatives of the deceased patients gave consent for the publication of the material related to the case study.

## References

[CR1] Bäuerle J, Fischer A, Hornig T, Egger K, Wengenmayer T, Bardutzky J (2013). Therapeutic hypothermia in cerebral air embolism: a case report. SpringerPlus.

[CR2] Finsterer J, Stöllberger C, Bastovansky A (2010). Cardiac and cerebral air embolism from endoscopic retrograde cholangio-pancreatography. Eur J Gastroenterol Hepatol.

[CR3] Han SS, Kim SS, Hong HP, Lee SY, Lee SJ, Lee BK (2010). Massive paradoxical air embolism in brain occurring after central venous catheterization: a case report. J Korean Med Sci.

[CR4] Heckmann JG, Ganslandt O (2004). Images in clinical medicine. The Mount Fuji sign. N Engl J Med.

[CR5] Heckmann JG, Lang CJ, Kindler K, Huk W, Erbguth FJ, Neundörfer B (2000). Neurologic manifestations of cerebral air embolism as a complication of central venous catheterization. Crit Care Med.

[CR6] Koster GT, Brandsma D, Boot H, Kruyt ND (2012). Multiple cerebral air emboli during upper gastrointestinal endoscopy. J Neurol Neurosurg Psychiatry.

[CR7] Muth CM, Shank ES (2000). Gas embolism. New Engl J Med.

[CR8] Nern C, Bellut D, Husain N, Pangalu A, Schwarz U, Valavanis A (2012). Fatal cerebral venous air embolism during endoscopic retrograde chaolangiopancreatography – case report and review of th literature. Clin Neuroradiol.

[CR9] O’Dowd LC (2013). Air embolism: UpToDate.

[CR10] Pandurangadu AV, Paul JAP, Barawi M, Irvin CB (2012). A case report of cerebral air embolism after esophagogastroduodenoscopy: diagnosis and management in the emergency department. J Emerg Med.

[CR11] Valentino R, Hilbert G, Vargas F, Gruson D (2003). Computed tomographic scan of massive cerebral air embolism. Lancet.

[CR12] Van Hulst RA, Klein J, Lachmann B (2003). Gas embolism: pathophysiology and treatment. Clin Physiol Funct Imaging.

